# Supplementary material for: Correlated Dynamics in Egocentric Communication Networks
Source: PLoS One. 2012 Jul 12;7(7):e40612. doi: 10.1371/journal.pone.0040612 (PMC3395632; doi:10.1371/journal.pone.0040612)
Supplement: Text S1 — Properties of single link bursty behaviour. (PDF) [file pone.0040612.s001.pdf]

Correlated dynamics in egocentric communication networks
Supplementary Information

M. Karsai, K. Kaski and J. Kertész

May 18, 2012

We have shown in the main text that bursty communication event trains are more likely to evolve on single links rather than between a larger group of people. However, this behaviour can be influenced by other trivial processes or mediated by technology related constraints. Here we are presenting results of additional analysis to support the significance of our findings and to verify that the evolution of single link bursty trains is a true feature of human communication behaviour. In the following in Section 1 we present a null model study while in Section 2 we apply systematic filtering to our results.

1 Significance of single link bursty behaviour

From the results presented in the main text we concluded that correlated bursty trains of mobile calls and SMS events usually evolve on single links between two connected people and that they are not the results of the communication of a larger social group. However, to show the significance of this observation yet we need to compare the real sequences to reference model sequences where we eliminate the effect of the actual correlations. To define such a null model system we randomly shuffle the receivers of calls of each user and calculate the same characteristic functions on the shuffled sequences. As we do not change the timing of any event, in the null model sequences the same bursty trains can be detected as in the original sequences and also the degrees of the individuals remain the same. However, since the receivers were shuffled, the trains do not necessarily evolve between the ego and one neighbour but are possibly distributed between other neighbours of the actual user. This way we can cease out the effect of correlation causing the evolution of bursty trains towards a single neighbour thus enabling us to see how strong influence it plays on the observed behaviour.

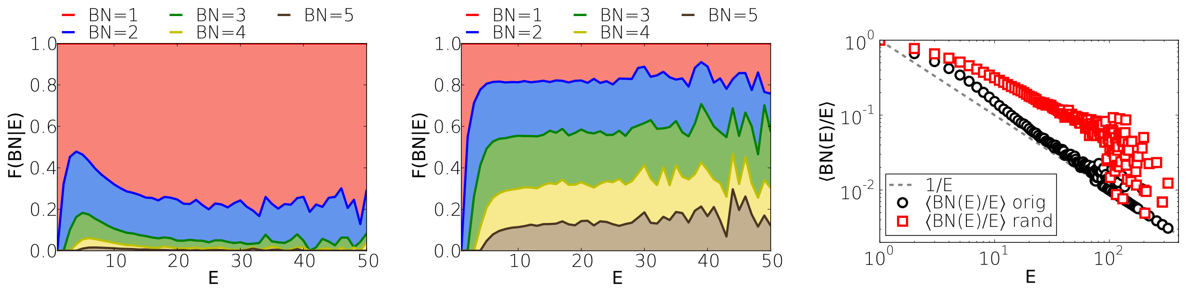

(a) $F(BN|E)$ orig (b) $F(BN|E)$ rand (c) $\langle BN/E \rangle$

Figure 1: Fraction of outgoing mobile call trains with size E induced by an ego towards $BN = 1$ (red),2 (blue),3 (green),4 (yellow) and 5 (brown) number of bursty neighbours in the (a) original and (b) randomly shuffled sequences (for definition see text). (c) Average ratio of BN number of bursty friends and the E size of the actual train detected in the original (black circles) and null model (red squares) sequences.

We measure the same quantities for the null model as for the original sequences in the main text. The effect of the removed correlations is straightforward if we compare Fig.1.a and b where the fraction of outgoing trains evolving between the ego and different number of bursty neighbours is presented ((a)

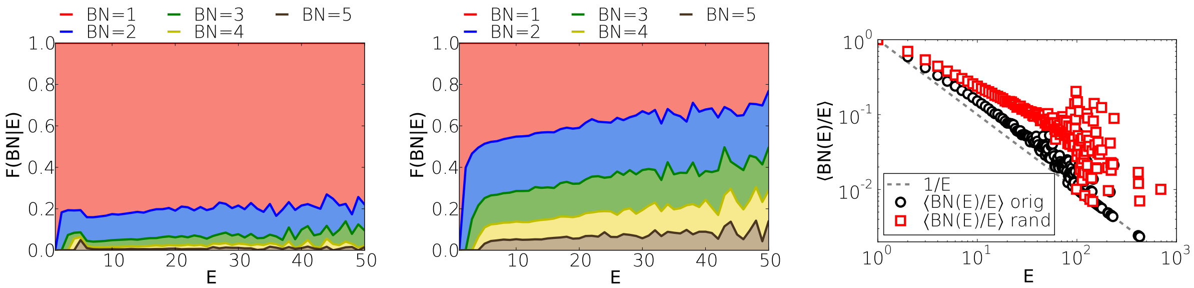

(a) $F(BN|E)$ orig (b) $F(BN|E)$ rand (c) $\langle BN/E \rangle$

Figure 2: Fraction of outgoing SMS trains with size E induced by an ego towards $BN = 1$ (red),2 (blue),3 (green),4 (yellow) and 5 (brown) number of bursty neighbours in the (a) original and (b) randomly shuffled sequences (for definition see text). (c) Average ratio of BN number of bursty friends and the E size of the actual train detected in the original (black circles) and null model (red squares) sequences. Note that in random case longer SMS trains could evolve as multi-part SMS' were distributed between several users and were detected as independent events.

for original and (b) for randomly shuffled sequences). In the original case $\sim 80\%$ of trains evolve between dyads while only a considerably smaller fraction is directed towards a larger number of people. In the random case the contrary is true as the fraction of trains between dyads turns out to be around $\sim 20\%$ while the fraction of trains evolving between the ego and larger BN number of friends is considerably increased. The same effect is visible if we look at the average ratio $\langle BN(E)/E \rangle$ of the BN number of people called by a single user in a train of length E (see Fig.1.c). In the main text we mentioned that this ratio is $1/E$ if only one individual was called in a train of size E. We can confirm such scaling behaviour for trains in the original sequence (black symbols), however, this is not true for trains in the null model sequences where the ratio is much larger (red symbols) assigning several people connected in a single train. We repeated all these measurement for SMS sequences after we generated the corresponding null model sequences. The results shown in Fig.2 reflect very similar asymptotic behaviour as for voice calls and demonstrates the strong effect of correlations which cause the single link bursty SMS trains evolution observed in the original sequences.

This way we demonstrated the significance of the observed phenomena as in the original sequences bursty trains are more likely to evolve between pairs of individuals than in randomly shuffled sequences. One can quantify the strength of the effect by comparing the original and randomly shuffled results showing considerably different behaviour.

2 Trivial dependences of single link bursty behaviour

The evolution of bursty trains on single links is a significant feature of human communication as we demonstrated in the previous section. However, it can be an artifact partially or completely due to some topological features or being mediated by trivial technological constraints. In this section we discuss the influence of these effects to show how much the observed behaviour depends upon them.

2.1 Degree dependence of single link bursty behaviour

One trivial topological constraint which can influence the evolution of single link bursty trains comes from the degree heterogeneity of the backgrounding social network. We know that its structure shows a broad degree distribution [1] indicating a large number of users with degree $k = 1$. If a user has only one friend then obviously any train induced by him/her can evolve only just on a single link. To check whether the degree heterogeneity is the reason behind the single link trains we performed a systematic analysis where we recalculated the $F(BN|E)$ fractions after we removed nodes with degree $k < k_{\geq}$.

The results are depicted in Fig.3.a for mobile phone calls (Fig.4.a for SMS) where the fractions calculated for $BN = 1, 2, 3, 4$ and 5 bursty neighbour are shown (red, blue, green, yellow and brown areas accordingly) for nodes with degree larger or equal than $k_{\geq} = 1, 2, 4$ or 8. Here the lighter the color

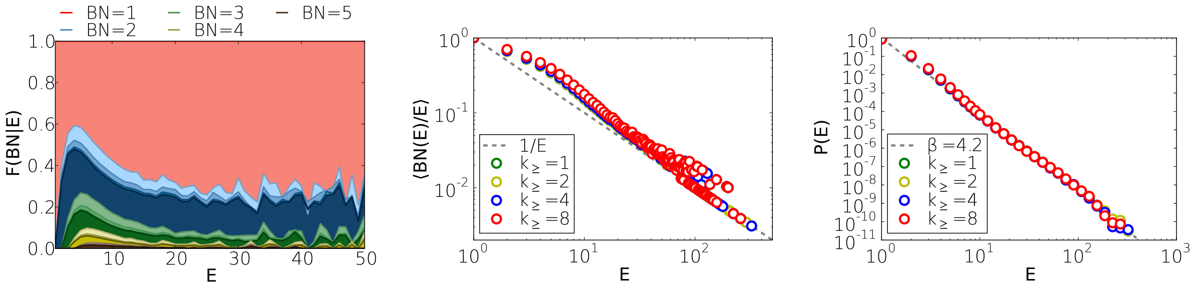

(a) $F(BN|E)$ (b) $\langle BN/E \rangle$ (c) $P(E)$

Figure 3: Fraction of outgoing mobile call trains with size E induced by an ego towards $BN = 1$ (red), 2 (blue), 3 (green), 4 (yellow) and 5 (brown) number of bursty neighbours after removing nodes with degree $k < k_{\geq}$. The lighter the color shade the higher the corresponding $k_{\geq}$ value. (b) Average ratio of BN number of bursty friends and the E size of the actual train detected for nodes with degree $k \geq k_{\geq}$. (c) Distribution of train size in call sequences of nodes with degree $k \geq k_{\geq}$. The fitted straight line assigns a power-law function with exponent $\beta = 4.2$.

the higher the corresponding $k_{\geq}$ value. It is straightforward that by removing larger and larger degree nodes the fraction of trains evolving on single links (denoted by the red area) is slightly decreasing, but their majority remains in all cases. Also minor differences were found after calculating the $\langle BN(E)/E \rangle$ ratios for different $k_{\geq}$ values (see Fig.3.b for call and Fig.4.b for SMS sequences) and the corresponding $P(E)$ distributions. These functions show quite robust behaviour against degree filtering (see Fig.3.c 4.c). Thus we can conclude that degree heterogeneities cannot be the main reason behind the evolution of single link bursty trains as by removing small degree nodes the observed behaviour does not change considerably.

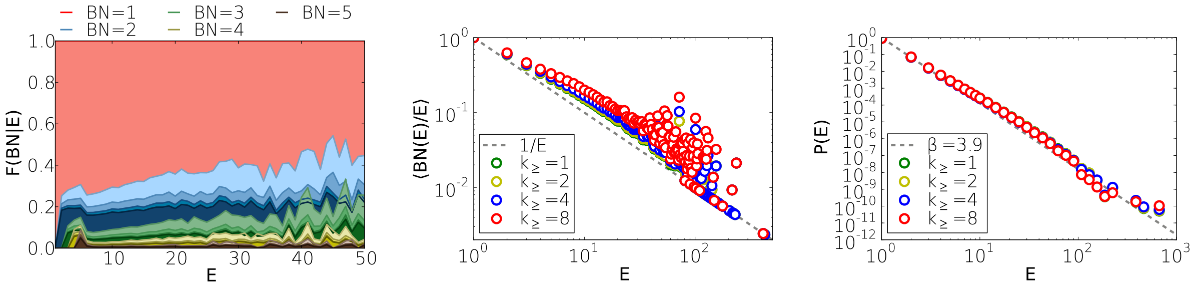

(a) $F(BN|E)$ (b) $\langle BN/E \rangle$ (c) $P(E)$

Figure 4: Fraction of outgoing mobile SMS trains with size E induced by an ego towards $BN = 1$ (red), 2 (blue), 3 (green), 4 (yellow) and 5 (brown) number of bursty neighbours after removing nodes with degree $k < k_{\geq}$. The lighter the color shade the higher the corresponding $k_{\geq}$ value. (b) Average ratio of BN number of bursty friends and the E size of the actual train detected for nodes with degree $k \geq k_{\geq}$. (c) Distribution of train size in SMS sequences of nodes with degree $k \geq k_{\geq}$. The fitted straight line assigns a power-law function with exponent $\beta = 3.9$.

2.2 Duration dependence of single link bursty behaviour

A technology related problem for voice calls could be also a possible source of the observed single link bursty train evolution. When the caller initiate a call it is possibly picked up by the called person, however it can reach also an answering machine. In this case the caller possibly repetitively calls again several times, which behaviour appears like a bursty pattern including a series of rather short calls. This phenomena is a possible reason why longer trains tend to build up of shorter calls as we see in Fig.5, where the cumulative distribution $P_{\sum}(d(E))$ of call duration for calls detected in trains with size E is shown. Unfortunately we have no information to make a distinction whether a call was picked up by

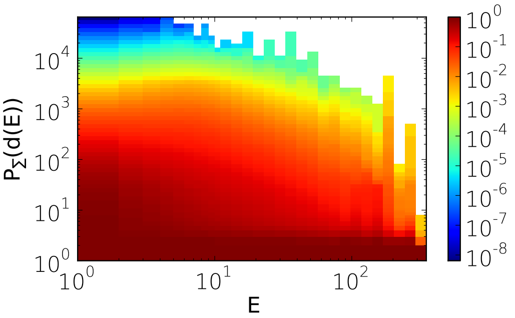

Figure 5: Cumulative distribution of duration of calls in bursty trains of length E.

an answering machine or answered by the call receiver, but we can study the effect of short calls which possibly contain most of the answering machine events.

We measured the same characteristic functions as in Section 2.1 but here instead of removing users with certain degrees we removed calls with duration shorter than $d_{\geq}$. As we see in Fig.6.a the fraction of trains containing events with duration $d \geq d_{\geq} = 1, 2, 3, 4$ or 5 second towards different number of bursty neighbours did not change considerably. This is also true for the corresponding $\langle BN(E)/E \rangle$ ratios and $P(E)$ distributions. However, one effect is visible in all these figure as by removing short calls some longer trains brake up to shorter sequences increasing the probability of smaller E sizes and decreasing the probability of longer trains.

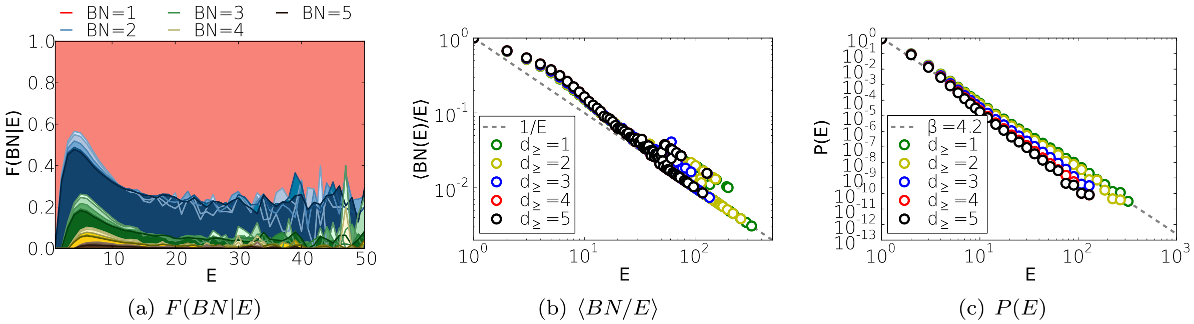

(a) $F(BN|E)$ (b) $\langle BN/E \rangle$ (c) $P(E)$

Figure 6: Fraction of outgoing mobile call trains with size E induced by an ego towards $BN = 1$ (red), 2 (blue), 3 (green), 4 (yellow) and 5 (brown) number of bursty neighbours after removing events with duration $d < d_{\geq}$. The lighter the color shade the higher the corresponding $d_{\geq}$ value. (b) Average ratio of BN number of bursty friends and the E size of the actual train detected for events with duration $d \geq d_{\geq}$. (c) Distribution of train size in call sequences with events having duration $d \geq d_{\geq}$. The fitted straight line assigns a power-law function with exponent $\beta = 4.2$.

As we stated earlier we cannot decide about a call whether it was carrying information or just hanged up after it reached an answering machine. Nevertheless, the above analysis indicates that even by removing all short calls, which might contain answering machine events, the behaviour of the characteristic measures do not change considerably. Thus we can safely conclude that answering machine mediated communication events are not the main reason for the evolution of the single link bursty trains.

References

[1] Onnela JP et.al. (2007) Analysis of a large-scale weighted network of one-to-one human communication. *New. J. Phys.* 9:179.
